# AI-Assisted Diagnosis of *Trichomonas vaginalis* from Routine Gram-Stained Vaginal Smears

**DOI:** 10.3390/diagnostics16121763

**Published:** 2026-06-08

**Authors:** Fernando Ernesto Ortega-Ojeda, Daniella Peña-Pedraza, Manuel Linares-Rufo, Francisco-Javier Bueno-Guillén, Álvaro Irigoyen-von-Sierakowski, Carlos García-Bertolín, Harold Bermúdez-Marval, José-Manuel Gómez-Pulido

**Affiliations:** 1 Departamento de Ciencias de la Computación, Universidad de Alcalá, Ctra. Madrid–Barcelona Km 33.6, 28871 Alcalá de Henares, Madrid, Spainfjavier.bueno@uah.es (F.-J.B.-G.); jose.gomez@uah.es (J.-M.G.-P.); 2 Departamento de Microbiología, Hospital Universitario Príncipe de Asturias (HUPA), 28805 Alcalá de Henares, Madrid, Spain; manuellinares@fundacionio.com (M.L.-R.); airigoyenvs@gmail.com (Á.I.-v.-S.); carlos.gbcb@gmail.com (C.G.-B.); haroldbermudez3@gmail.com (H.B.-M.); 3 Ramón y Cajal Institute for Health Research (IRYCIS), 28034 Madrid, Spain

**Keywords:** *Trichomonas vaginalis*, artificial intelligence, computer-aided diagnosis, Gram staining, vaginal smears, clinical microbiology, image analysis, diagnostic support

## Abstract

**Background/Objectives**: *Trichomonas vaginalis* is one of the most prevalent non-viral sexually transmitted infections worldwide. Although Gram staining is routinely performed in clinical microbiology laboratories for the evaluation of vaginal samples, it is not considered a diagnostic method for *T. vaginalis*, which represents a missed diagnostic opportunity in routine practice. This study aimed to evaluate an artificial intelligence (AI)-assisted diagnostic approach for the identification of *T. vaginalis* directly from routine Gram-stained vaginal smears. **Methods**: A retrospective dataset of Gram-stained vaginal smear images was analysed using a cascaded AI-based framework combining image processing and classification. The image selection and quality control were performed under the supervision of a specialised clinical microbiologist. All cases were independently confirmed by polymerase chain reaction (PCR), which served as the reference diagnostic standard. Model performance was assessed using standard diagnostic metrics, including accuracy, sensitivity, specificity, area under the receiver operating characteristic curve (AUC), Cohen’s kappa, and Matthews correlation coefficient (MCC). Held-out independent testing was used to assess generalisability beyond the internal validation subset. **Results**: The proposed AI-assisted approach demonstrated high diagnostic performance for the identification of *T. vaginalis*, achieving an AUC of 0.973, Cohen’s kappa of 0.87, and an MCC of 0.87. The system showed high diagnostic concordance with PCR results across both internal and external validation datasets, supporting the feasibility and reproducibility of the approach under routine laboratory conditions. **Conclusions**: This study shows that artificial intelligence may enhance the diagnostic utility of routinely performed Gram-stained vaginal smears by enabling reliable identification of *T. vaginalis*. The proposed approach could be integrated into standard microbiology workflows as an objective decision-support or triage adjunct, facilitating early identification and supporting clinical decision-making without altering existing laboratory procedures.

## 1. Introduction

*Trichomonasvaginalis* is a flagellated protozoan and the causative agent of trichomoniasis, the most prevalent nonviral sexually transmitted infection worldwide, with an estimated annual incidence of 170–190 million cases [1]. Despite its high prevalence and its association with significant clinical complications—including vaginitis, cervicitis, infertility, adverse pregnancy outcomes, and increased susceptibility to human immunodeficiency virus (HIV) infection—trichomoniasis remains frequently underdiagnosed, particularly in low-resource healthcare settings [1,2]. The burden of infection is disproportionately higher in women, reaching prevalences of up to 22% in sexually transmitted infection clinics, whereas in men prevalence rarely exceeds 5%, with infections often remaining asymptomatic [1].

The diagnosis of *T. vaginalis* traditionally relies on wet mount microscopy, culture, and nucleic acid amplification tests (NAATs). Wet mount microscopy is rapid and inexpensive but has limited sensitivity (35–70%), is highly dependent on parasite motility, and is strongly affected by delays between sample collection and examination [2,3]. Culture improves sensitivity (44–88%) but requires prolonged incubation and specialised laboratory conditions. NAATs are currently considered the reference standard owing to their high sensitivity (76–100%); however, their implementation is frequently constrained by cost, infrastructure requirements, and limited availability in many clinical and resource-constrained settings [2,3].

Gram staining remains a core component of routine clinical microbiology and is systematically integrated into standard laboratory workflows for the evaluation of vaginal exudates and other clinical specimens [4,5]. Owing to its rapid turnaround time, low operational cost, and minimal infrastructure requirements, it continues to serve as a first-line diagnostic tool in hospital laboratories worldwide. However, it is not routinely used for the diagnosis of *T. vaginalis*. The principal limitation arises from staining-induced morphological alterations that obscure characteristic features of the parasite, complicating its differentiation from leukocytes, epithelial cells, and staining arteifacts. In Gram-stained smears, *T. vaginalis* typically appears as a 10–20 µm ovoid structure with absent flagella and granular cytoplasm, closely resembling host cellular elements. This reduced morphological distinctiveness diminishes diagnostic confidence and contributes to its under-recognition in routine practice. Nonetheless, retrospective analyses have demonstrated that *T. vaginalis* can be identified in Gram-stained samples, including cases initially reported as negative by wet mount microscopy [6], indicating that diagnostic information is present but not consistently recognized.

Advances in artificial intelligence have enabled robust automated analysis of microscopy images across diverse microbiological applications. High-performance detection and segmentation of *T. vaginalis* have been reported using fluorescence microscopy, wet mount videos, and phase-contrast imaging [7,8,9]. However, despite the widespread and standardised implementation of Gram staining in clinical laboratories [4,5], automated detection of *T. vaginalis* from Gram-stained smears has not been specifically investigated. Leveraging artificial intelligence within this established, low-cost diagnostic framework could therefore enhance detection without modifying existing laboratory infrastructure.

Against this background, this study aimed to develop and evaluate a transferable and scalable artificial intelligence-assisted diagnostic support system for the detection of *Trichomonas vaginalis* in Gram-stained vaginal smears. Although Gram staining is not routinely used for the diagnosis of trichomoniasis, it represents a widely available and standardised technique in clinical microbiology laboratories, making it an attractive substrate for automated diagnostic support. The proposed approach is designed to operate on routinely acquired microscopy images, is compatible with conventional laboratory microscopes and smartphone-based imaging, and does not require additional reagents or specialised infrastructure. By leveraging artificial intelligence to support the interpretation of an already established but diagnostically underutilised technique, this work aims to enhance diagnostic accessibility, reproducibility, and efficiency in routine clinical microbiology, particularly in settings where access to molecular diagnostics is limited.

**Figure 1:**
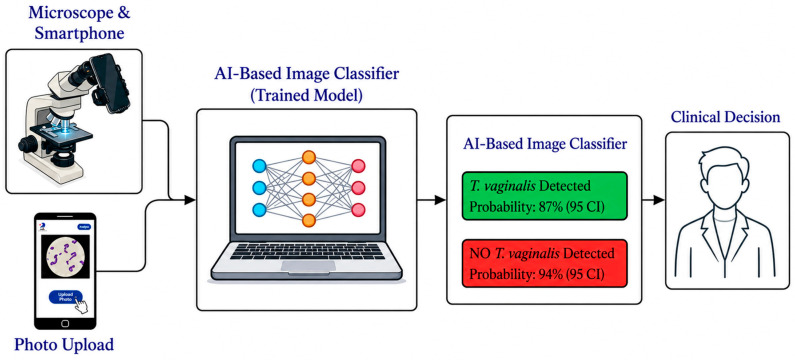
Conceptual overview of the AI-assisted diagnostic workflow. The figure illustrates the intended integration of the trained classification model into a mobile-assisted microscopy setting. System architecture details are shown for contextual purposes only.

Figure 1 illustrates the conceptual workflow of the proposed AI-assisted diagnostic support system, showing how Gram-stained vaginal smear images acquired through conventional or smartphone-assisted microscopy are processed to generate probabilistic classification, while clinical decision-making remains under medical supervision.

## 2. Materials and Methods

### 2.1. Study Design and Clinical Specimens

This retrospective observational study analysed Gram-stained vaginal smear images collected between 2024 and 2025 from female patients attending the Príncipe de Asturias University Hospital (HUPA), Alcalá de Henares, Spain.

The samples were processed according to the routine Gram-staining protocol used in the clinical microbiology laboratory. The microscopy images were obtained from routinely stained diagnostic preparations, reflecting the variability expected under standard laboratory conditions rather than idealised experimental staining.

Image acquisition was performed in the Department of Clinical Microbiology under a formal collaboration agreement with the Health Computing and Intelligent Systems (HCIS) research group.

All samples were fully anonymised prior to analysis, and no identifiable personal data were accessible to the research team. Each microscopy image corresponded to a routinely prepared Gram-stained vaginal smear obtained as part of standard clinical care, ensuring that all analysed images originated from a standardised and widely implemented diagnostic workflow in routine clinical microbiology.

For each clinical specimen, representative microscopic fields were initially selected and captured by an experienced clinical microbiologist during routine evaluation. *T. vaginalis* infection status was established using the Abbott Alinity m STI Assay (Abbott Molecular Inc., Des Plaines, IL, USA), a multiplex real-time nucleic acid amplification test (NAAT) targeting *T. vaginalis*-specific nucleic acid sequences, performed on the Alinity m platform according to the manufacturer’s instructions and routine laboratory protocol. The molecular result was used as the reference standard for evaluating the AI-based image classification model.

### 2.2. Gram Staining and Image Acquisition

Microscopy images were acquired using a conventional optical microscope (Leica DM3000 LED, Leica Microsystems GmbH, Wetzlar, Germany) at 100× magnification. To assess compatibility with low-cost imaging solutions, a smartphone camera (Samsung Galaxy S24, Samsung Electronics Co., Ltd., Suwon, Republic of Korea) was mounted on the microscope eyepiece for image capture. All images were stored in Portable Network Graphics (PNG) format, a lossless image standard widely supported by mobile devices and commonly used for native image acquisition and storage in smartphone-based systems. This choice ensures preservation of fine morphological details while maintaining compatibility with cost-effective, mobile-assisted microscopy workflows, facilitating future deployment in resource-constrained clinical settings.

### 2.3. Image Preprocessing and Field-of-View Standardisation

Original micrographs were acquired at high resolution (approximately 3000 × 3000 pixels). To ensure uniformity across samples and remove peripheral artifacts, a standardised field-of-view (FOV) extraction procedure was applied prior to model training and inference.

Each image underwent linear contrast scaling to facilitate boundary delineation of the circular microscope field. The enhanced images were converted to grayscale and subjected to binary thresholding to enable contour detection. The largest connected contour was assumed to correspond to the circular FOV, and a minimum enclosing circle was computed. A circular mask was then generated to isolate the microscope field.

Pixels outside the detected FOV were replaced with a uniform black background to avoid introducing artificial edge artefacts. The masked region was subsequently cropped using the bounding coordinates of the enclosing circle.

All cropped images were resized to 760 × 770 pixels to match the input resolution used for model training and inference. The entire processed image was used for classification without region-level annotation.

No data augmentation techniques were applied. The only transformations performed for model input preparation were resizing, tensor conversion, and intensity normalisation.

Differences in the image quality between microscopes may affect the AI performance by introducing domain shift. Variations in illumination, contrast, focus, optical resolution, colour balance, sensor response, and image-acquisition conditions can modify the visual features used by the model for classification. In this study, the images were acquired under routine conditions, including smartphone-assisted microscopy, and were standardised by resizing and normalisation before the training. Nevertheless, these preprocessing steps may not fully remove microscope-dependent variability. Therefore, future prospective implementation should include harmonised acquisition protocols and external validation using images obtained with different microscopes and acquisition settings. If the performance degradation is observed, robustness may be improved through training datasets balanced across microscope types, colour and intensity augmentation, local fine-tuning, calibration, or domain-adaptation approaches.

### 2.4. AI Model Development and Training

Several neural network options were explored during the initial model-development stage, including ResNet-based models and different members of the EfficientNet family [10]. Based on these preliminary exploratory tests, together with the reported efficiency and transfer-learning performance of EfficientNet architectures, EfficientNetV2-XL pretrained on ImageNet-21K was selected as the final backbone for the image-classification workflow. The model was implemented in the PyTorch 2.8.0+cu128 (with CUDA 12.8 support) framework, using pretrained weights obtained from the TIMM library [11]. EfficientNetV2-XL was selected because of its balance between representational capacity and computational efficiency, which is suitable for high-resolution microscopy images. The original classification head was replaced by a task-specific binary classification head adapted to the detection of *T. vaginalis*.

The dataset was initially partitioned by reserving 15% of the images (380 images) as an independent test set, which remained completely unseen throughout model development and hyperparameter tuning. This independent test subset comprised 206 micrographs corresponding to the *T. vaginalis* (CatAB) category and 174 micrographs corresponding to the negative (CatN) category.

The remaining 85% of the dataset (2160 images) was used for model training and internal validation. This subset comprised 1157 micrographs belonging to the CatAB class and 1003 micrographs belonging to the CatN class. From this pool, 1836 images were allocated to the training set and 324 images to the validation set. Stratified sampling was applied to preserve the original class distribution across all data splits.

The model was trained and evaluated at the image level. The images were assigned to training and validation subsets using a stratified splitting strategy based on the class labels. Because multiple images may originate from the same patient, sample, or slide, future studies should implement strict patient-, sample-, or slide-level grouping during the dataset partitioning to prevent potential data leakage between training, validation, and test sets.

The model training was performed using the AdamW optimiser(PyTorch Foundation, Linux Foundation, Wilmington, DE, USA). During the fine-tuning, differential learning rates were applied: 1 × 10^−5^ for the trainable backbone layers and 1 × 10^−4^ for the classification head. A weight decay of 1 × 10^−4^ was used, and the learning rate was further controlled using a cosine annealing scheduler with a minimum learning rate of 1 × 10^−6^. The training was conducted for 50 epochs with a batch size of 16, and data loading was parallelised using 8 workers.

**Figure 2:**
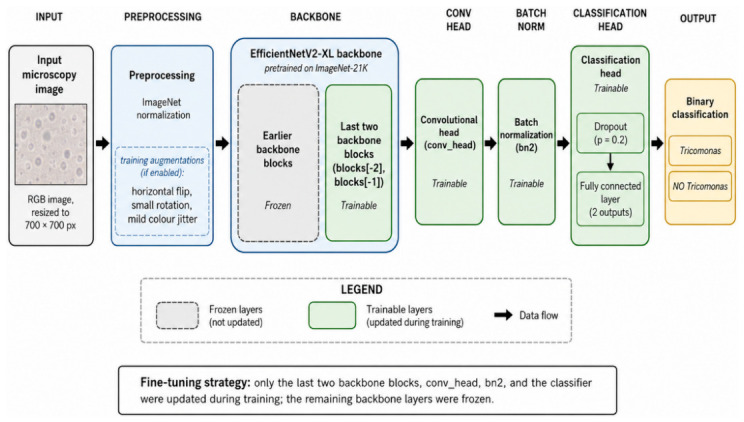
Schematic representation of the EfficientNetV2-XL-based network architecture used for binary classification. Created with the assistance of AI.

To improve reproducibility, Table 1 provides a layer-by-layer summary of the model configuration and fine-tuning strategy, and Figure 2 shows a schematic representation of the EfficientNetV2-XL network architecture. Briefly, the input microscopy images were resized to 700 × 700 pixels and normalised before being processed by the EfficientNetV2-XL backbone. During the fine-tuning, only the last two backbone blocks, the convolutional head, the final batch-normalisation layer, and the classification head were trainable, whereas the remaining backbone layers were frozen. The classification head consisted of a dropout layer followed by a fully connected layer with two output units.

All experiments were conducted in a Python 3.10 environment on a workstation equipped with an Intel i9-14900K CPU, 128 GB RAM, and an NVIDIA RTX 4090 GPU, running Windows 11 with CUDA 12.1 support.

### 2.5. Performance Evaluation Metrics

The time to result depends primarily on the pre-analytical and image-acquisition steps rather than on the AI inference itself. In this study, the model was trained and evaluated using previously acquired microscopy images; therefore, the complete end-to-end workflow time, including the sample preparation, microscopy examination, image capture, and AI classification, was not formally benchmarked. Once an image is available, the computational classification step is rapid and is not expected to represent the limiting stage of the workflow. Future prospective implementation should include a formal measurement of the full time-to-result under routine laboratory conditions.

Diagnostic performance was assessed using standard classification metrics, including accuracy, precision, sensitivity (recall), specificity, F1-score, Cohen’s kappa coefficient, and Matthews correlation coefficient (MCC). Macro-averaged and weighted-average metrics were computed to account for class imbalance.

Receiver operating characteristic (ROC) curves and the corresponding area under the curve (AUC) were generated to evaluate discrimination performance across decision thresholds. Confusion matrices and prediction confidence distributions were analysed to characterise error patterns and model behaviour.

Calibration performance was additionally evaluated using Expected Calibration Error (ECE), Maximum Calibration Error (MCE), and Brier score. Temperature scaling was applied prior to calibration assessment.

### 2.6. Statistical Analysis

Statistical analyses were conducted using Python 3.11 with SciPy (version 1.11) and scikit-learn (version 1.4).

Point estimates were first computed for discrimination and calibration metrics. The multiclass Brier score was calculated as the mean squared difference between predicted probabilities and one-hot encoded ground truth labels. Negative log-likelihood (NLL) was computed using the log-loss formulation. Expected calibration error (ECE) and maximum calibration error (MCE) were calculated using a top-1 confidence approach, with predictions grouped into 15 equally spaced confidence bins.

To estimate the uncertainty of calibration metrics, bootstrap resampling was applied. Specifically, sample indices were resampled with replacement while preserving the original sample size. For each bootstrap replicate, Brier score, NLL, ECE, and MCE were recalculated. A total of 300 bootstrap iterations were performed, and 95% confidence intervals were derived using the percentile method (2.5th and 97.5th quantiles). These intervals are reported as calibration bootstrap confidence intervals.

For discrimination metrics (accuracy, precision, sensitivity), 95% confidence intervals were calculated using the Wilson score method for binomial proportions. For composite metrics, including Cohen’s kappa, Matthews correlation coefficient (MCC), and weighted F1-score, confidence intervals were estimated using multinomial bootstrap resampling with 10,000 iterations.

In addition to global calibration confidence intervals, uncertainty within reliability diagrams was quantified at the bin level. Accuracy per bin was estimated with Wilson confidence intervals, while mean confidence per bin was accompanied by confidence intervals of the mean calculated using a t-distribution.

All confidence intervals were computed at a significance level of 0.05.

### 2.7. Use of Generative Artificial Intelligence

Generative artificial intelligence tools were not used to generate research data, microscopy images, diagnostic labels, analytical results, statistical outputs, or model predictions in this study. No generative artificial intelligence systems were employed for study design, data interpretation, or result generation. AI-based tools were used only to assist with the preparation of schematic figures (Figure 1 and Figure 2), troubleshooting during the model-development coding phase, and grammar and language editing. All scientific content, analyses, interpretations, and conclusions were reviewed and approved by the authors.

### 2.8. Scope of the Present Study and System Integration Context

The present study focuses exclusively on the development, training, and evaluation of the artificial intelligence model for binary image-level classification of *T. vaginalis* presence in Gram-stained vaginal smears, including the estimation of prediction confidence. System-level components such as cloud infrastructure, data storage, application interfaces, and clinical integration workflows are part of a broader ongoing translational project and are therefore outside the scope of this article.

Within that broader framework, the AI model described here constitutes the core analytical component intended for future integration into mobile-assisted microscopy workflows and clinical decision-support pipelines. However, the current manuscript is intentionally limited to the methodological validation of the algorithmic approach and its diagnostic performance.

## 3. Results

### 3.1. Model Training and Internal Validation

#### 3.1.1. Training and Validation Curves

**Figure 3:**
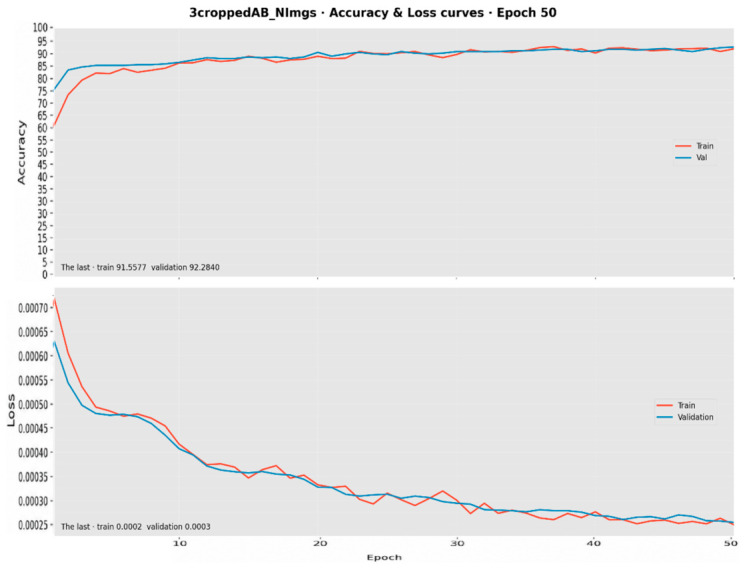
Evolution of training and validation accuracy (**top**) and training and validation loss (**bottom**) during 50 epochs of fine-tuning of the EfficientNetV2-XL architecture. These curves summarise the model optimisation during the training and should not be interpreted as diagnostic sensitivity or specificity. Convergence was observed with closely aligned training and validation performance at the final epoch.

The fine-tuning process was conducted over 50 epochs. Training and validation accuracy increased progressively during the initial training phase and stabilised after approximately epoch 20 (Figure 3).

At epoch 50, training accuracy reached 91.56%, while validation accuracy reached 92.28%.

Training and validation loss decreased during early epochs and reached a plateau towards the end of training. Final loss values were 0.0002 for training and 0.0003 for validation.

The present model was designed as a binary classifier focused specifically on *T. vaginalis* detection and does not provide multiplex information on co-infections or other common causes of vaginitis or sexually transmitted infections. Therefore, the proposed AI-assisted Gram-stain workflow should not be interpreted as a replacement for multiplex NAAT panels, which remain necessary when simultaneous detection of multiple pathogens is clinically required. Its current clinical positioning is more appropriately considered as a rapid, low-cost triage or screening adjunct for *T. vaginalis* detection in routine microscopy workflows, particularly in symptomatic vaginitis settings or where molecular testing is limited, delayed, or selectively used. Future developments could explore multi-class or multi-label AI models incorporating additional pathogens or microscopy findings, but these would require larger annotated datasets and prospective validation against established multiplex molecular workflows.

#### 3.1.2. Quantitative Validation Performance

Diagnostic performance was evaluated with the independent validation set, which was not used for weight updates during training but was used for model monitoring and selection.

On this dataset (*n* = 324 images), performance was assessed at epoch 50. The fine-tuned EfficientNetV2-XL model achieved an overall accuracy of 92.28% (95% CI: 0.8886–0.9472). Sensitivity for the detection of *T. vaginalis* (CatAB) reached 94.32% (95% CI: 0.8986–0.9688), while specificity for negative samples (CatN) was 89.86%. The positive predictive value (precision) for CatAB was 91.71%. Detailed class-specific precision, recall, and F1-score values with 95% confidence intervals are reported in Table 2.

The discordant cases were defined as those in which the AI-based classification did not match the PCR result. For analytical purposes, PCR was retained as the reference standard; therefore, the AI-positive/PCR-negative cases were considered false positives, whereas AI-negative/PCR-positive cases were considered false negatives. The discordant cases were reviewed by the clinical microbiology team to identify possible morphological or technical factors associated with misclassification.

**Table 2:** Diagnostic performance metrics per class with 95% confidence intervals (CI) on the validation subset. CatAB denotes *T. vaginalis*-positive images and CatN denotes *T. vaginalis*-negative images.

Class	Precision [95% CI]	Recall [95% CI]	F1-Score [95% CI]
CatAB	0.9171 [0.8678, 0.9491]	0.9432 [0.8986, 0.9688]	0.9300 [0.9022, 0.9566]
CatN	0.9301 [0.8761, 0.9616]	0.8986 [0.8395, 0.9376]	0.9141 [0.8773, 0.9458]

In addition to conventional diagnostic indicators, global agreement metrics were calculated. The model achieved a Cohen’s kappa of 0.8441 (95% CI: 0.7836–0.8937) and a Matthews correlation coefficient (MCC) of 0.8445 (95% CI: 0.7796–0.9041). The balanced accuracy was 0.9209 (95% CI: 0.8887–0.9474).

At the aggregate level, the macro F1-score was 0.9220 (95% CI: 0.8902–0.9534) and the weighted F1-score was 0.9227 (95% CI: 0.8931–0.9506).

### 3.2. Overall Diagnostic Performance on the Independent Test Set

The model was evaluated on an independent test set comprising 380 Gram-stained vaginal smear images that were not used during training or validation.

Overall accuracy was 0.900 (95% CI: 0.866–0.926).

For CatAB (*n* = 206), precision was 0.893 (95% CI: 0.844–0.927), recall was 0.927 (95% CI: 0.883–0.955), and F1-score was 0.910 (95% CI: 0.878–0.935).

For CatN (*n* = 174), precision was 0.910 (95% CI: 0.856–0.945), recall was 0.868 (95% CI: 0.810–0.910), and F1-score was 0.888 (95% CI: 0.850–0.923).

The macro-averaged F1-score was 0.899 (95% CI: 0.867–0.928), and the weighted F1-score was 0.900 (95% CI: 0.871–0.930). Cohen’s kappa coefficient was 0.798 (95% CI: 0.731–0.860), and the Matthews correlation coefficient (MCC) was 0.799 (95% CI: 0.734–0.859). Detailed class-specific performance metrics with 95% confidence intervals are summarised in Table 3.

**Figure 4:**
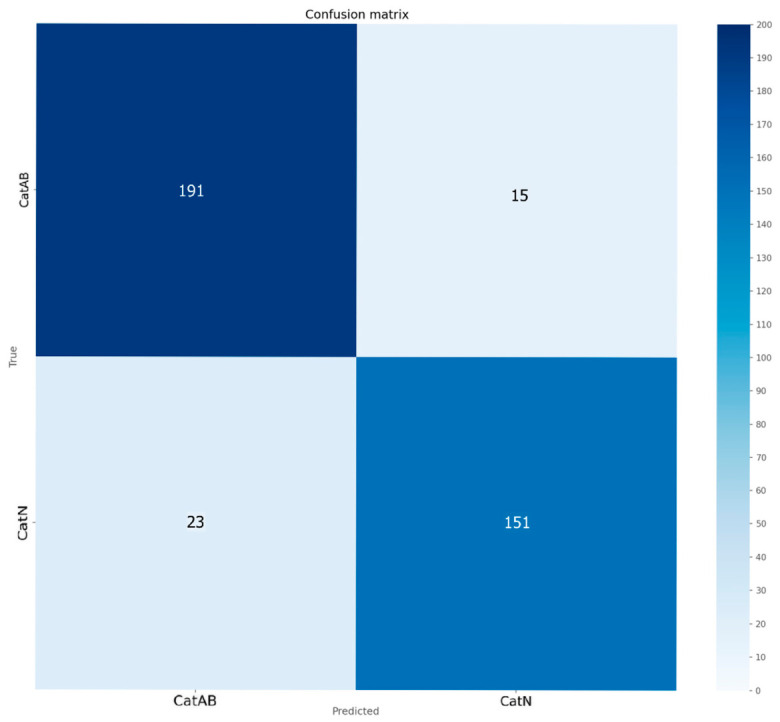
Confusion matrix for the independent test dataset. Rows represent ground-truth PCR labels, and columns represent model predictions. CatAB denotes *T. vaginalis*-positive images and CatN denotes *T. vaginalis*-negative images.

The confusion matrix obtained from the independent test dataset is presented in Figure 4. The model correctly classified 191 positive samples (true positives) and 151 negative samples (true negatives). Misclassifications included 15 false negatives and 23 false positives.

### 3.3. Class-Specific Discrimination Performance and ROC Analysis

Receiver operating characteristic (ROC) analysis was performed to evaluate the threshold-independent discriminatory capacity of the model on the independent test dataset (*n* = 380).

For the positive class (CatAB), the ROC curve demonstrated separation between infected and non-infected specimens, with an area under the curve (AUC) of 0.973 (95% CI: 0.959–0.984).

Similarly, for CatN, the AUC was 0.973 (95% CI: 0.959–0.985) under the one-versus-rest (OvR) evaluation scheme.

**Figure 5:**
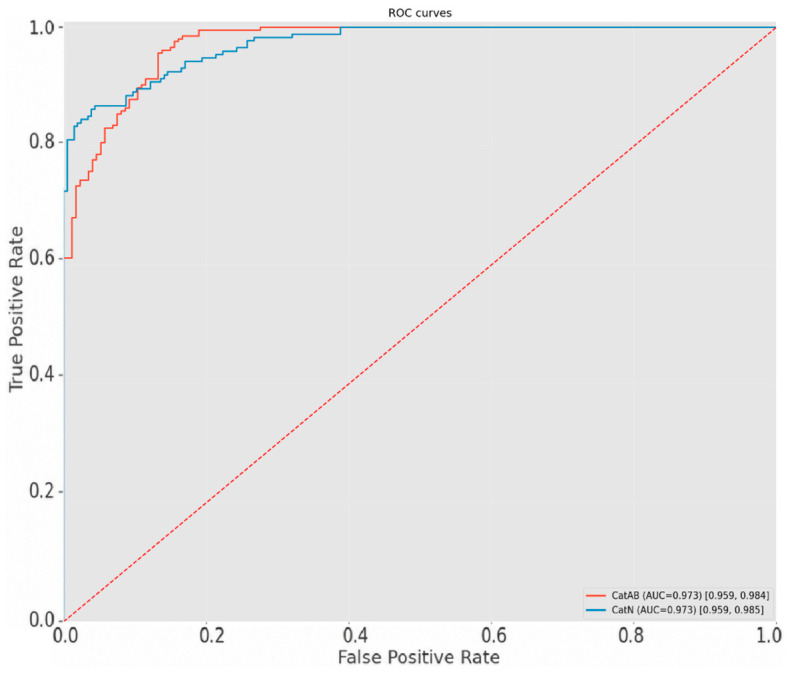
Receiver operating characteristic (ROC) curves for CatAB and CatN on the independent test dataset (*n* = 380). The area under the curve (AUC) was 0.973 (95% CI: 0.959–0.984) for CatAB and 0.973 (95% CI: 0.959–0.985) for CatN. The dashed diagonal line represents the no-discrimination reference line, corresponding to random classification performance (AUC = 0.5).

The corresponding ROC curves are presented in Figure 5.

### 3.4. Prediction Probability Distribution

The distribution of predicted Top-1 probabilities was analysed in the independent test dataset. Correctly classified samples were predominantly associated with higher predicted probability values for their respective classes, whereas misclassified samples were more frequently observed in proximity to the decision threshold.

**Figure 6:**
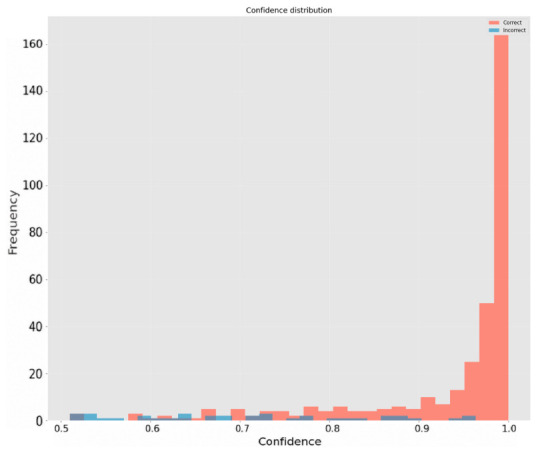
Distribution of predicted Top-1 probabilities in the independent test dataset.

As illustrated in Figure 6, the majority of predictions were concentrated at high confidence levels (>0.80), with a substantial proportion exceeding 0.90. Lower-confidence predictions were less frequent and corresponded more often to classification errors.

### 3.5. Calibration and Reliability of Probabilistic Outputs

Calibration on the independent test dataset after temperature scaling (T = 0.6000) yielded a multiclass Brier score of 0.131 (95% CI: 0.101–0.167) and a negative log-likelihood (NLL) of 0.209 (95% CI: 0.169–0.262). The expected calibration error (ECE) computed using a Top-1 confidence binning strategy with 15 bins was 0.023 (95% CI: 0.022–0.058), while the maximum calibration error (MCE) was 0.235 (95% CI: 0.148–0.521).

**Figure 7:**
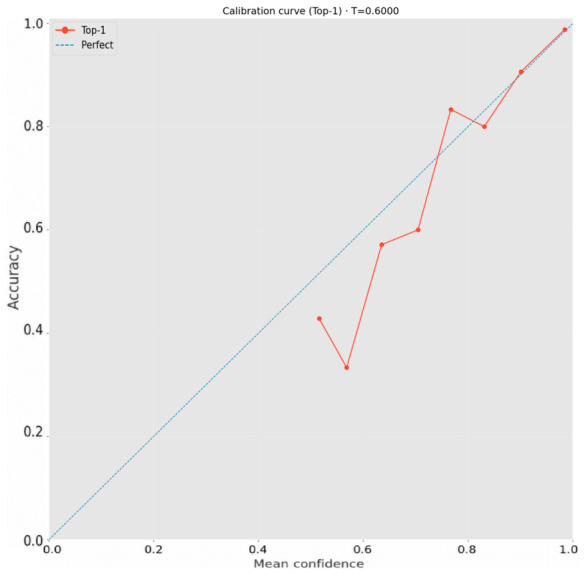
Global calibration curve (Top-1) on the independent test dataset after temperature scaling (T = 0.6000).

The global Top-1 calibration curve (Figure 7) demonstrated near-diagonal alignment at higher confidence levels, with moderate deviations observed in mid-confidence intervals.

**Figure 8:**
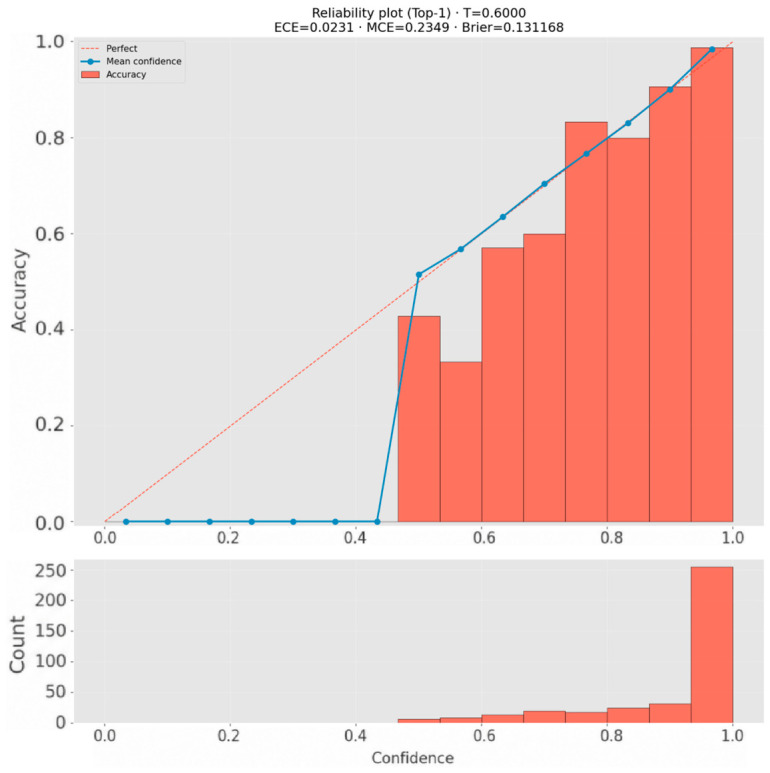
Reliability diagram (Top-1) showing bin-wise accuracy, mean confidence, and confidence distribution.

The reliability diagram (Figure 8) further illustrates the relationship between mean predicted confidence and observed accuracy across bins, together with the distribution of prediction confidences.

## 4. Discussion

### 4.1. Diagnostic Performance and Consistency Across Independent Testing

The diagnosis of *T. vaginalis* remains operationally challenging in routine practice. Wet mount microscopy is highly operator-dependent and limited by rapid loss of motility, whereas permanent staining methods, including Gram staining, may obscure characteristic morphological features such as flagella or the undulating membrane [12,13]. Although NAATs represent the molecular reference standard, their implementation may be constrained by cost, infrastructure, and turnaround time.

The present study evaluated whether diagnostically actionable information can be reproducibly extracted from Gram-stained vaginal smears using deep learning, with predefined training, validation, and independent test partitions.

The diagnostic sensitivity and specificity were calculated from the final classification outputs using the molecular result as the reference standard. These diagnostic metrics are distinct from the training and validation accuracy curves shown in Figure 3, which describe the model optimisation across epochs.

The transition from internal validation (accuracy 92.28%) to independent testing (accuracy 90.0%) reflects a modest absolute reduction of approximately 2.3 percentage points. However, interpretation of performance shifts must consider inferential uncertainty. The 95% confidence intervals for global accuracy, balanced accuracy, and F1-scores demonstrated substantial overlap between validation and independent test subsets. This overlap supports statistical stability and argues against meaningful degradation beyond expected sampling variability.

Sensitivity remained consistently high (94.3% validation vs. 92.7% test), while specificity showed a moderate reduction (89.9% to 86.8%). Importantly, this asymmetric behaviour did not produce disproportionate collapse in agreement metrics. The Matthews correlation coefficient (MCC) and Cohen’s kappa remained within the substantial agreement range under independent testing, indicating preserved joint behaviour of true positives, true negatives, and error components.

From a diagnostic safety perspective, the preservation of sensitivity under external conditions is particularly relevant, as false-negative *T. vaginalis* results may delay therapy and facilitate transmission. The observed performance profile therefore supports robust case detection while maintaining acceptable specificity.

The review of the discordant cases suggested several possible sources of false-positive and false-negative classifications. The false-positive AI outputs may be favoured by inflammatory debris, epithelial cell fragments, mucus, staining artefacts, or background structures that partially resemble parasite morphology. Conversely, false-negative classifications may occur in samples with low presumed parasite burden, poorly preserved organisms, weak staining, partial field representation, or suboptimal focus. Staining heterogeneity and differences in the image quality may also affect the feature extraction by the model. These findings suggest that the discordant classifications are not necessarily random, but may reflect biologically and technically challenging microscopic fields. Future studies should include prospective assessment of discordant cases and, where possible, model refinement using larger datasets enriched with morphologically difficult examples.

Taken together, these findings are consistent with generalisation stability rather than validation-specific optimisation.

### 4.2. Discrimination Stability and Threshold-Independent Evaluation

Receiver operating characteristic analysis confirmed that ranking capacity was maintained under independent evaluation. Preservation of AUC indicates that discriminative ordering of positive versus negative samples was not confined to the operating threshold selected during testing.

The convergence of macro-F1, weighted-F1, MCC, and kappa across validation and test datasets further supports robustness. MCC is particularly informative in binary diagnostic tasks because it incorporates all four confusion matrix elements and remains stable under moderate class imbalance. The preservation of MCC under external testing reduces the likelihood that performance is driven by prevalence artefacts.

From an inferential standpoint, the absence of non-overlapping confidence intervals between validation and independent metrics suggests that performance differences do not reach statistical significance under bootstrap estimation. This strengthens the interpretation that generalization was preserved within expected variance bounds.

### 4.3. Structured Error Behaviour and Morphological Ambiguity

The independent confusion matrix revealed relatively fewer false negatives than false positives, accounting for higher sensitivity than specificity. This error pattern is plausible in Gram-stained vaginal smears, where epithelial debris, leukocytes, inflammatory exudate, bacterial aggregates, and staining heterogeneity may partially resemble parasite morphology under digital magnification [12,13]. The absence of motility cues further increases structural ambiguity compared with wet mount microscopy.

Importantly, analysis of predicted probability distributions demonstrated that misclassified samples were predominantly concentrated near the decision threshold, whereas correctly classified cases clustered in high-confidence regions (commonly >0.80 and frequently >0.90). High-confidence incorrect predictions were uncommon. This structured topology suggests that residual errors arise primarily from intrinsically ambiguous morphologies rather than indiscriminate, overconfident misclassification. In practice, such behaviour is preferable because it preserves interpretability and supports controlled threshold adjustment.

### 4.4. Calibration Quality and Probabilistic Reliability

For clinical decision support, discrimination must be complemented by probabilistic reliability. Following temperature scaling, the model exhibited low expected calibration error (ECE) and acceptable Brier score and negative log-likelihood values on the independent test set, indicating that predicted confidence values were, on average, aligned with empirical accuracy.

Reliability curves showed close alignment with the ideal reference line in high-confidence regions—where prediction density was greatest—while deviations were localised primarily within intermediate confidence bins. This pattern is coherent with the probability distribution findings and suggests that calibration deviations concentrate in the same ambiguity-dominated region where classification errors are most frequent.

Importantly, calibration refinement was achieved without degrading discrimination, preserving ranking performance while improving probability coherence. The joint preservation of MCC and low ECE supports the use of predicted probabilities for threshold adaptation or risk stratification, provided that operating points are selected according to intended clinical use.

### 4.5. Decision-Analytic Perspective and Threshold Flexibility

Because misclassifications cluster near the decision boundary and high-confidence errors are rare, the model permits threshold adaptation according to clinical priorities.

In screening-oriented settings, the decision threshold may be lowered to maximise sensitivity with limited impact on high-confidence specificity. Conversely, in confirmatory workflows, threshold elevation could reduce false positives without collapsing discrimination performance, given preserved AUC behaviour.

The combination of stable discrimination, low ECE, and structured probability distribution supports threshold-dependent optimisation rather than rigid binary deployment.

### 4.6. Positioning Within AI-Assisted Microbiology

Automation in microbiology has demonstrated value in accelerating organism detection and standardising interpretation. Platforms such as the Accelerate PhenoTest BC Kit and BioFire systems exemplify integration of algorithmic support in diagnostic workflows [14,15].

Parallel studies have explored AI-based analysis of vaginal specimens using digital culture imaging [16] or CNN-based recognition of discharge samples [17]. However, many approaches rely on acquisition modalities not universally standardised across laboratories.

By focusing on conventional Gram-stained vaginal smears—already embedded in routine workflows—and validating against a molecular reference standard, this study extends AI-assisted analysis to a globally accessible diagnostic substrate.

Model deployment is computationally lightweight, with inference latency compatible with real-time microscopic evaluation, supporting potential integration in resource-limited or edge-based environments.

### 4.7. Limitations and External Validity

This study has some limitations related to the retrospective nature of the sample collection. The images were obtained from routine clinical practice in women undergoing vaginal microbiological evaluation, mainly because of suspected vaginitis or sexually transmitted infection. Consequently, the cohort should be interpreted as a clinically referred population rather than as a population-based screening cohort. This may introduce a selection bias, since symptomatic women or women with clinical suspicion of infection may be overrepresented. In addition, complete symptom-based information was not consistently available for all cases, preventing a reliable stratification of the dataset into symptomatic and asymptomatic groups. Therefore, the prevalence observed in this cohort may differ from that expected in general screening populations, and predictive values may vary when the model is applied in settings with different disease prevalence. Future prospective validation should include broader and more representative populations, complete demographic and clinical metadata, and explicit stratification according to the symptom status.

The staining and imaging variability also represent potential limitations for the AI-based microscopy analysis. Although the images used in this study were obtained from routine clinical preparations and therefore included realistic intra-laboratory variability, all samples were processed within the same institution and diagnostic workflow. Differences between laboratories in staining protocols, staining intensity, colour balance, fixation, smear thickness, background staining, decolourisation, slide preparation, microscope type, and acquisition hardware may affect the visual appearance of the microscopic fields and introduce domain shift [18]. Such variability could influence the model performance, particularly if the staining or imaging characteristics differ substantially from those represented in the training data. Therefore, future multicentre validation should include images obtained using different staining protocols, laboratories, microscopes, and acquisition systems to assess robustness and generalisability.

Unlike wet mount microscopy, the AI-based approach evaluated in this study is not dependent on parasite motility or immediate microscopic examination, because classification is performed on acquired images from stained preparations. This may reduce one of the main operational limitations of conventional wet mount examination. Nevertheless, AI performance remains dependent on the quality of the pre-analytical and imaging workflow. Factors such as sample adequacy, parasite burden, uneven organism distribution on the slide, smear thickness, fixation and staining quality, inflammatory debris, focus, illumination, and image resolution may affect the visual features available to the model and influence classification performance. Therefore, future implementation should include standardised procedures for sample collection, smear preparation, staining, field selection, and image acquisition.

Several model-related limitations should also be considered. The model was developed as an image-level classifier. If multiple images from the same patient, sample, or slide are distributed across different dataset partitions, data leakage may occur and performance may be overestimated. Although the present study supports the feasibility of AI-assisted microscopy for *T. vaginalis* detection, future studies should apply strict patient-, sample-, or slide-level separation between training, validation, and test sets.

In addition, the model classified whole microscopy images, and parasite-level annotation or segmentation was not performed. Therefore, it cannot be concluded with certainty that the model relied exclusively on *T. vaginalis* morphology. Indirect cues, including staining characteristics, background texture, inflammatory debris, focus, illumination, or slide quality, may also have contributed to the predictions. Future work should include visual explainability methods, such as Grad-CAM, together with region-level annotation or segmentation, to verify whether the most discriminative image regions correspond to parasite-like structures.

Another limitation is that a formal head-to-head comparison with alternative architectures was not performed. Although preliminary exploratory tests were conducted during the model development, the final analysis focused on EfficientNetV2-XL and did not systematically compare its performance with ResNet, DenseNet, MobileNet, Vision Transformer architectures, or other classical machine-learning approaches. Therefore, the present results should not be interpreted as demonstrating the superiority of EfficientNetV2-XL over all alternative models. Rather, they should be interpreted as evidence of feasibility. Future studies should include systematic benchmarking of multiple architectures, including lightweight models suitable for clinical deployment, and should compare AI-based classification with conventional expert microscopy in prospective clinical settings.

A further limitation is the absence of multiplexing capability. The current model performs binary classification for *T. vaginalis* detection and does not identify co-infections or other pathogens. Accordingly, it should be positioned as a rapid, low-cost triage adjunct rather than as a standalone replacement for multiplex NAAT panels. Future work should investigate multi-class or multi-label extensions using larger annotated datasets and prospective comparison with multiplex molecular testing.

A further limitation concerns the difference in the effective sampling volume between microscopy-based image analysis and molecular testing. PCR/NAAT protocols generally process a larger volume of specimen or transport medium and therefore capture nucleic acid from a broader fraction of the biological sample. In contrast, Gram-stained microscopy is based on a small aliquot spread across a 2D smear, and the AI model analyses a limited standardised field of view after the image cropping. Consequently, the analysed image may not fully represent the whole slide or specimen.

This 2D sampling constraint may reduce the real-world clinical sensitivity in cases with low parasite burden, uneven organism distribution, poor smear representation, or suboptimal field selection. The issue may be particularly relevant in low-prevalence or asymptomatic screening populations, where organisms may be sparse and the probability of capturing them in a single microscopic field is lower. Therefore, the reported sensitivity should be interpreted in the context of the image-based workflow evaluated here and relative to the molecular reference standard, rather than as evidence that single-field AI-assisted microscopy provides the same sampling depth as PCR/NAAT. Future implementations should consider multi-field imaging per slide, standardised field-selection protocols, and sample-level prediction based on aggregation of multiple image-level outputs.

The proposed AI-assisted workflow should also be interpreted as a human-in-the-loop decision-support system rather than as a fully autonomous diagnostic replacement. Real-world implementation would still require trained laboratory personnel for residual quality-control steps, including assessment of smear adequacy, representative-field selection, verification of image quality, and review of low-confidence or discordant AI outputs. Therefore, the estimated reduction in manual workload should be interpreted cautiously. Net labour savings will depend on the balance between time saved during routine screening and the time required for residual human quality control, as well as on local staffing models and wage structures. Future implementation should evaluate hybrid human–AI triage protocols prospectively, including AI confidence thresholds, review rates, error costs, staff time, and implementation costs across different healthcare systems.

Despite these limitations, the preservation of discrimination, agreement metrics, and calibration under independent evaluation provides a strong methodological foundation for future external validation studies.

### 4.8. Potential Economic Impact and Clinical Relevance

From a health-economic perspective, the proposed AI-assisted Gram-staining approach may have the potential to reduce diagnostic costs and optimise laboratory workload. However, the estimates presented here should be interpreted as an exploratory budget-impact scenario rather than as a formal cost-effectiveness analysis. They are based on local clinical microbiology experience and approximate costs within the Spanish healthcare context, and are intended to illustrate the potential scale of economic impact rather than to provide definitive evidence of cost saving.

The implementation of an AI-assisted diagnostic framework for *T. vaginalis* may address an important gap in routine clinical microbiology. Although Gram staining is a core, low-cost component of standard laboratory workflows, its use for the identification of *T. vaginalis* has traditionally been limited by stain-induced morphological alterations that obscure characteristic features such as flagella, making it difficult for laboratory staff to distinguish the parasite from leukocytes or epithelial cells. The proposed AI system may help address this limitation by providing automated screening that extracts diagnostically relevant information from routine stained smears without requiring any modification of existing procedures.

Within the Spanish National Health System (SNS), this approach could potentially offer efficiency gains if implemented within an appropriately validated diagnostic workflow. The public cost of a microbiological PCR test in Spain ranges between €44.90 and €65.00, whereas the additional estimated cost associated with AI-assisted interpretation of an already available Gram-stained smear would be substantially lower, approximately €0.50 per image or analysis. These figures should be interpreted as approximate local estimates rather than universal costs. Absolute savings are expected to be highly jurisdiction-specific and may vary according to PCR/NAAT costs, staff costs, reimbursement structures, laboratory workflow, confirmatory testing algorithms, and implementation costs. In healthcare systems where molecular testing is substantially more expensive, such as some North American public or private laboratory settings, the potential economic benefit of an AI-assisted triage strategy could be greater, although this would require local prospective health-economic validation.

As an illustrative scenario, for a standard Spanish hospital serving approximately 250,000 inhabitants, the potential annual economic impact could reach around €205,000 under the assumptions described above. This estimate combines several hypothetical sources of saving. First, optimisation of the laboratory staff time could account for approximately €58,000, assuming a reduction of around 5 min of manual workload per sample and an effective hourly staff cost of approximately €28–32 within the SNS. Second, around €72,000 could potentially be saved by reducing the need for confirmatory PCR testing in selected diagnostic pathways, assuming approximately 3000 PCR tests were avoided or redirected. Third, indirect savings could arise from earlier detection and treatment, potentially reducing complications such as pelvic inflammatory disease (PID), estimated here at approximately €45,000. Finally, a reduction in follow-up consultations and associated logistical costs could account for approximately €30,000. These estimates are intended to illustrate a plausible order of magnitude rather than to establish confirmed savings.

The economic estimates presented in this study should therefore be interpreted with caution. They represent an exploratory scenario based on approximate local costs and routine clinical microbiology experience, rather than a prospective health-economic evaluation. The analysis did not formally measure real-world workflow times, implementation costs, confirmatory testing pathways, or the downstream clinical and economic consequences of false-positive and false-negative AI classifications. In particular, false-negative results could delay diagnosis and treatment, whereas false-positive results could lead to unnecessary confirmatory testing, treatment, follow-up, or patient anxiety. Therefore, although the proposed approach may have potential to reduce laboratory workload and PCR-related costs, definitive conclusions regarding cost savings require prospective workflow-based validation in routine clinical settings.

Future studies should include formal health-economic assessment incorporating laboratory workflow, staff time, confirmatory testing algorithms, prevalence-dependent predictive values, model error costs, patient outcomes, implementation costs, and jurisdiction-specific cost structures across different healthcare settings.

## 5. Conclusions

This study demonstrates that diagnostically meaningful morphological information contained within conventional Gram-stained vaginal smears can be systematically extracted using deep learning for the detection of *T. vaginalis*. Across internal validation and held-out independent testing, discrimination performance remained stable, with preserved agreement metrics and high sensitivity under external evaluation. The substantial overlap of the confidence intervals between datasets supports consistent model behaviour rather than validation-specific optimisation.

Importantly, misclassifications were predominantly concentrated near the decision threshold, while high-confidence incorrect predictions were uncommon. Together with low expected calibration error following temperature scaling, these findings indicate that the model’s probabilistic outputs are not only discriminative but also numerically coherent and interpretable. This is essential for threshold adaptation and risk-informed deployment in clinical workflows.

Rather than replacing molecular diagnostics, the proposed system is best conceptualised as a decision-support or triage adjunct capable of operating on a globally implemented diagnostic substrate without requiring major modification of the existing laboratory infrastructure. With appropriate validation, optimisation, and workflow integration, this approach could support decentralised or resource-limited diagnostic settings.

Although multicentre prospective validation is warranted to confirm robustness under domain shift and to quantify real-world clinical impact, the convergence of discrimination stability, calibration reliability, and structured error behaviour provides strong evidence of technical rigour and translational potential.

Collectively, these results support the feasibility of AI-assisted analysis of Gram-stained vaginal smears as a reproducible, scalable, and clinically relevant approach for the detection of *T. vaginalis*.

## Figures and Tables

**Table 1:** Layer-by-layer summary of the model configuration.

Stage	Layer/Module	Configuration	Trainable During Fine-Tuning	Purpose/Output
Input	Microscopy image	RGB image resized to 700 × 700 pixels	Not applicable	Standardised image input for the neural network
Preprocessing	Tensor conversion and normalisation	ImageNet-compatible normalisation	Not applicable	Adapts input images to the pretrained backbone requirements
Backbone	EfficientNetV2-XL	Pretrained on ImageNet-21K	Partially trainable	Deep feature extraction from microscopy images
Backbone, early blocks	Earlier EfficientNetV2-XL backbone blocks	Original pretrained feature-extraction layers	No	Kept frozen to preserve general pretrained visual features and reduce overfitting
Backbone, final blocks	Last two backbone blocks, blocks [−2] and blocks [−1]	Final feature-extraction blocks of EfficientNetV2-XL	Yes	Domain-specific fine-tuning of high-level visual features
Convolutional head	conv_head	Final convolutional projection layer of the backbone	Yes	Refines the extracted feature representation before classification
Batch normalisation	bn2	Final batch-normalisation layer	Yes	Normalises the final feature representation
Classification head	Dropout	Dropout probability, *p* = 0.2	Yes	Reduces overfitting before the final classification layer
Classification head	Fully connected layer	Linear layer with two output units	Yes	Produces logits for the two output classes
Output	Binary classifier	Two-class output	Not applicable	Classification as *Trichomonas* or *NO Trichomonas*

**Table 3:** Diagnostic performance metrics per class with 95% confidence intervals (CI) on the test subset.

Class	Precision [95% CI]	Recall [95% CI]	F1-Score [95% CI]
CatAB	0.8925 [0.8439, 0.9273]	0.9272 [0.8833, 0.9554]	0.9095 [0.8782, 0.9347]
CatN	0.9096 [0.8563, 0.9445]	0.8678 [0.8095, 0.9103]	0.8882 [0.8504, 0.9233]

## Data Availability

The data supporting the findings of this study consist of anonymised Gram-stained microscopy images obtained from the Príncipe de Asturias University Hospital. Due to institutional and patient privacy regulations, these data are not publicly available. Access may be granted by the corresponding author upon reasonable request and approval by the hospital’s ethics committee.

## Abbreviations

The following abbreviations are used in this manuscript:

AI	Artificial Intelligence
AUC	Area Under the Receiver Operating Characteristic Curve
CatAB	*Trichomonas vaginalis*–positive class
CatN	*Trichomonas vaginalis*–negative class
CEIm	Ethics Committee for Drug Research
CI	Confidence Interval
CNN	convolutional neural network
HCIS	Health Computing and Intelligent Systems
HIV	Human immunodeficiency virus
HUPA	Príncipe de Asturias University Hospital
IRYCIS	Instituto Ramón y Cajal de Investigación Sanitaria
MCC	Matthews correlation coefficient
NAAT	nucleic acid amplification test
PCR	polymerase chain reaction
ROC	receiver operating characteristic
STI	sexually transmitted infection
TV	*Trichomonas vaginalis*
